# Clinical outcome of bioceramic sealer iRoot SP extrusion in root canal treatment: a retrospective analysis

**DOI:** 10.1186/s13005-022-00332-3

**Published:** 2022-08-31

**Authors:** Jing Li, Liuchi Chen, Chunmei Zeng, Yiwen Liu, Qimei Gong, Hongwei Jiang

**Affiliations:** 1 Stomatology Department, Shenzhen Hospital, Southern Medical University, Shenzhen, China; 2 Hospital of Stomatology, Guanghua School of Stomatology, Sun Yat-sen University and Guangdong Provincial Key Laboratory of Stomatology, 56 Ling Yuan Xi Road, Guangzhou, 510055 Guangdong China; 3 Department of Operative Dentistry and Endodontics, Hospital of Stomatology, Sun Yat-sen University, Guangzhou, China; 4 Department of Endodontics, Stomatological Hospital, Southern Medical University, Guangzhou, People’s Republic of China

**Keywords:** Root canal treatment, Single-cone technique, iRoot SP sealer, Extrusion, Clinical outcome\

## Abstract

**Background:**

During the obturation procedure, sealer extrusion occurs in some cases. iRoot SP is a kind of bioceramic sealer with superior physicochemical and biological properties. This article reports the outcome of iRoot SP extrusion in root canal treatment and the potential factors associated with the outcome.

**Methods:**

Ninety-nine patients and one hundred and eighty-five teeth treated between 2014 and 2020 were included in this retrospective study. All of the cases were filled with a single-cone technique and the iRoot SP sealer. The minimum follow-up visit period was 1 year. The outcome was evaluated by clinical examination and radiographic examination at recall and was classified as healed, healing (success), or not healed (failure).

**Results:**

The overall success rate of all teeth was 96.8%. The success rate of adequately filled teeth was 97.3%, while that of iRoot SP extrusion was 95.8%; the difference was not statistically significant. Factors such as gender, age, tooth position, follow-up visit period, size of periapical lesion, treatment type and extruding sealer amount had no influence on the outcome of iRoot SP extruded teeth.

**Conclusions:**

The results suggested that iRoot SP extrusion has no adverse effect on the outcome of root canal treatment, which may contribute to the endodontic treatment.

## Introduction

Root canal filling is the final procedure of root canal treatment and functions to densely seal the root canal system and the bacteria inside. The quality of filling is closely related to endodontic success. The optimal obturation is achieved by densely sealing and accurately stopping at the apical foramen [1].

Due to inflammatory destruction in the apical foramen or inappropriate root canal preparation from the operator, sealer extrusion occurs in some cases. Previous studies have suggested that extruded sealers or core materials might trigger inflammation, leading to worse outcomes than those cases in which the teeth are adequately filled [2, 3]. Histological examination demonstrated that overextended sealers cause severe inflammation in the short term. In the long run, there is no significant manifestation of infection around the extruded sealers, but proliferation of connective tissues and infiltration of several inflammatory cells [4].

In recent years, as dental materials have developed, bioceramic root canal sealers with good physicochemical and biological properties, such as mineral trioxide aggregate (MTA), have been introduced. Some researchers reported that extruded MTA has no adverse effect on periapical healing [5–8], while others held different opinions [9]. iRoot SP, similar to MTA, is also a kind of bioceramic root canal sealer, which composed of calcium silicate, calcium phosphate, zirconium oxide, calcium hydroxide and so on. It attracts clinical operators’ attention due to its superior properties. Specifically, iRoot SP is more biocompatible than AH Plus and MTA [10–17] and induces a mild inflammatory response in mouse subcutaneous tissue [18]. In addition, iRoot SP possesses an excellent apical sealing ability, which results in less apical microleakage [19–25]. After root canal obturation, it could also play a role in promoting osteogenesis [13, 16, 18, 26, 27] as well as continuously inhibiting bacteria, such as *Enterococcus faecalis*, *Candida albicans*, and *Staphylococcus aureus*, which may be related to its high alkalinity [28–32]. As a result, clinicians have come to a question whether over-obturated iRoot SP affects treatment outcomes. Therefore, this study aims to track our cases of iRoot SP over-obturation and evaluate the effect on periapical healing and tooth outcome.

## Material and methods

### Case selection and treatment procedure

Patients get the endodontic treatment in the Department of Operative Dentistry and Endodontics, Hospital of Stomatology affiliated to Sun Yat-sen University from 2014 through 2020. The protocol for this study was approved by the Medical Ethics Committee of Hospital of Stomatology, Sun Yat-sen University (approval number: KQEC-2020-05). Written informed consent were obtained from all subjects. The cases were selected or rejected according to the following criteria:

1. A completely developed tooth that demanded non-surgical root canal treatment or retreatment. The obturation was conducted using the single-cone technique with one gutta-percha and iRoot SP as the sealer.
2. Preoperative, postoperative, and follow-up radiographs and records documenting the treatments were well preserved.
3. The tooth was treated with acceptable obturation quality: all canals were prepared and filled within 1–2 mm of the radiographic apex. The gutta-percha was settled within the apical foramen.
4. The recall was for at least 1 year.
5. The tooth received adequate coronal restoration in a timely manner after root canal treatment.
6. The cases were rejected from this study if the following conditions occurred in the tooth: radiograph evidence of severe alveolar bone loss, perforation, cracks extending into canal orifices or vertical root fracture.

All of the selected teeth were treated by one specialist endodontist at two visits. Treatments began with local anesthesia and dental dam isolation. After access, the canal orifices were found under the dental microscope (OPMI PROergo, Zeiss, Oberkochen, Germany), and the working length was determined via an electronic apex locator (Raypex VI, VDW GmbH, Munich Germany). When the working length could not be confirmed by the electronic apex locator, a radiograph was taken. All of the canals were enlarged up to size 30 at least, which was 2–3 sizes larger than the initial canal size, by M3 Ni-Ti rotary instrumentation (Yirui Dental Group, Shanghai, China) using a standardized approach. In retreatment cases, previous obturation materials and canal obstructions were removed using a combination of ultrasonics and a ProTaper retreatment nickel-titanium system (Dentsply Maillefer, Ballaigues, Switzerland). The canals were irrigated with adequate 3% sodium hypochlorite and normal saline using a 31-G side-venting needle (Ultradent Products Inc., UT, US). After the preparation was complete, the canals were dried with paper points and dispensed with ApexCal (Ivoclar Vivadent AG, Schaan, Liechtenstein) as an intracanal medication. Cavition (GC, Tokyo, Japan) was used for temporary coronal sealing. Two weeks later, root canal obturation was performed at the second visit. The ApexCal was removed by both the master apical file and ultrasonics. iRoot SP was injected into the whole root canals, and gutta-percha cones that matched the master apical file were placed into the working length. Excess gutta-percha was cut, and the remaining gutta-percha was vertically packed with a plugger. For those irregular canals, additional cones were passively placed adjacent to the master cone. Excess sealer was removed from the chamber by ultrasonics. Finally, all of the teeth were sealed by permanent or temporary coronal restorations in a timely manner.

**Fig. 1:**
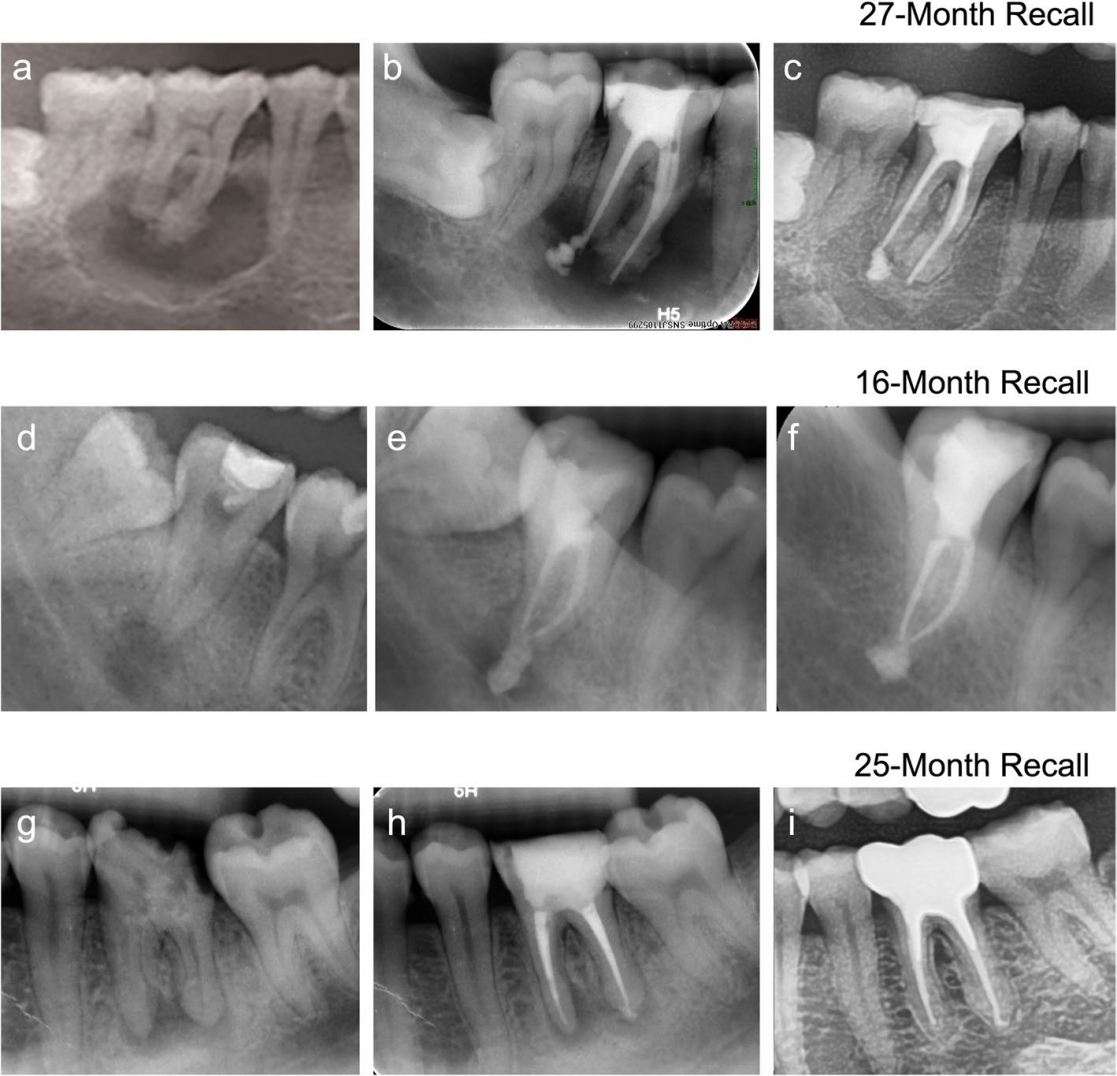
Preoperative (**a**, **d** and **g**), postoperative (**b**, **e** and **h**), and recall (**c**, **f** and **i**) radiographs of the outcomes of healed teeth

**Fig. 2:**
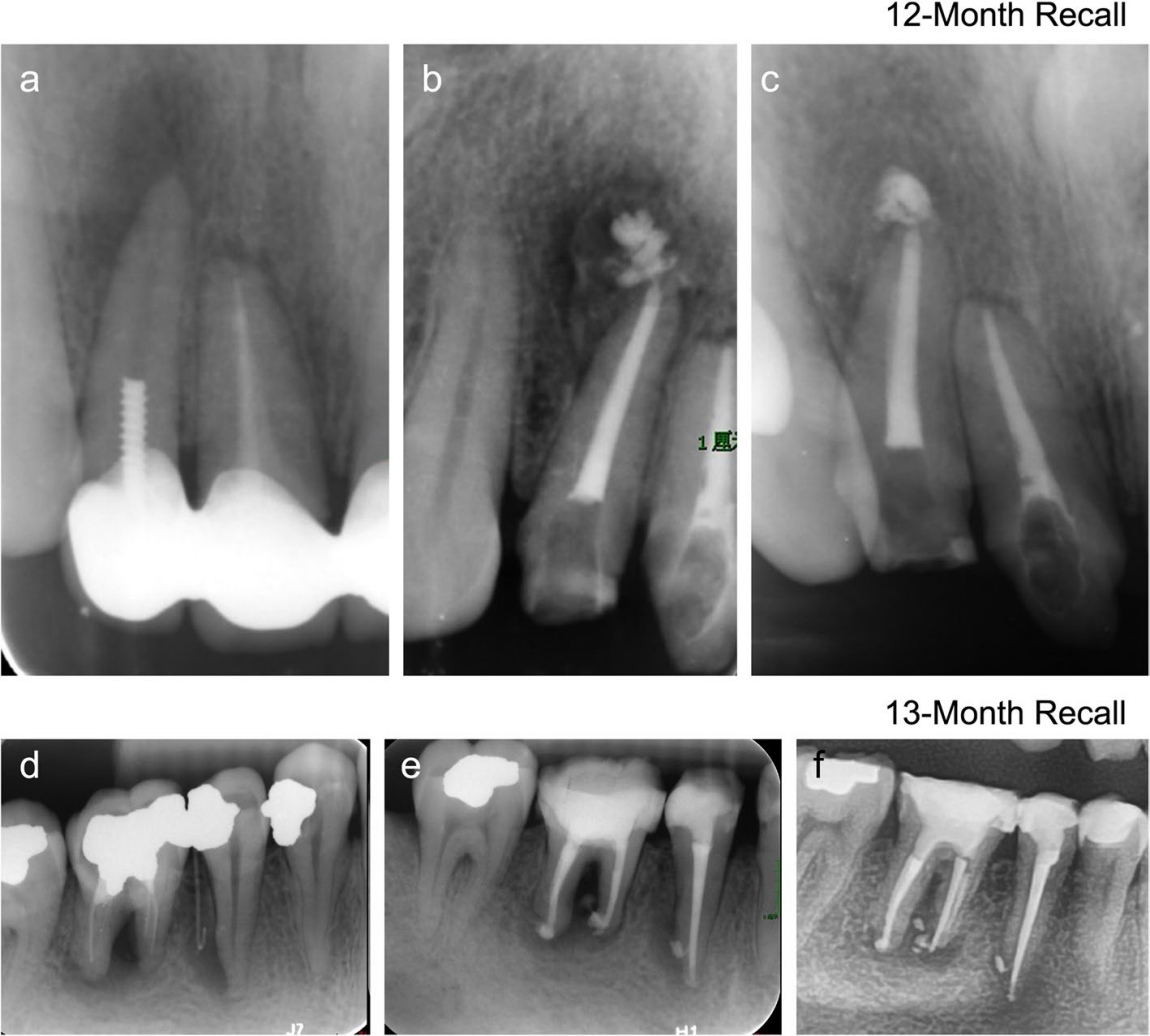
Preoperative (**a** and **d**), postoperative (**b** and **e**), and recall (**c** and **f**) radiographs of outcomes of healing teeth

**Fig. 3:**
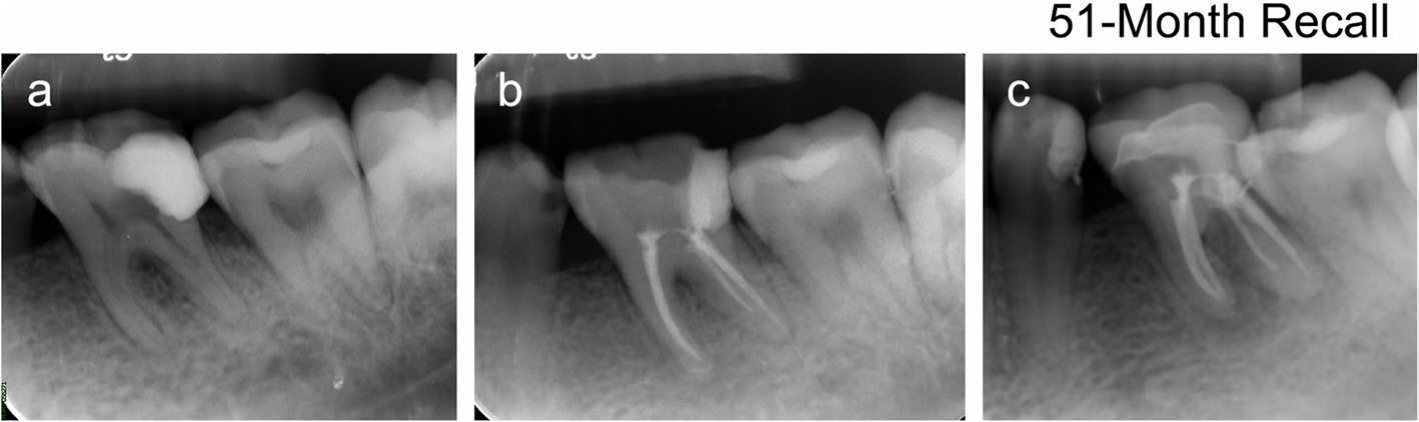
Preoperative (**a**), postoperative (**b**), and recall (**c**) radiographs of outcomes of unhealed teeth

Radiographic and clinical examinations were performed at recall appointments for the treated teeth. The presence of pain, a sinus tract, swelling, reaction to percussion and palpation, mobility and so on were all recorded. Preoperative, postoperative, and follow-up radiographs were evaluated by 2 examiners (not including the operator). The outcomes of the teeth were divided into the following 3 classifications:

1. Healed: Teeth function without any symptoms. Radiographs show no periapical radiolucency.
2. Healing: Teeth are asymptomatic and functional. Radiographs show that periapical lesions still exist but are smaller than before.
3. Non-healed: Teeth fail to function with symptoms, regardless of periapical radiolucency or asymptomatic teeth with unchanged, new, or enlarged periapical lesions. Examples of each outcome category are shown in Figs. 1, 2 and 3.

### Outcome assessment

The three outcome classifications were divided into two groups. Both healed and healing teeth were considered successful cases, and non-healed teeth were considered failure cases. To evaluate the factors that affected the outcome of the sealer-extruded teeth, we analyzed several possible variables, including the patients’ age, gender, tooth type, tooth position, presence of periapical lesion, lesion size, initial treatment or retreatment, follow-up time and extruding sealer amount.

### Data analysis

For statistical analysis, Fisher’s exact test were used to analyze the effect of each prognostic factor after the data were grouped. A *P* value < 0.05 was considered significant, and all tests were 2-sided. Statistical tests were performed with SPSS v25.0 software (IBM Corp, Armonk, NY).

## Results

Ninety-nine patients (twenty-one males and seventy-eight females) and one hundred and eighty-five teeth, which consisted of 61.1% teeth without sealer extrusion (113/185) and 38.9% teeth with sealer extrusion (72/185), were included for analysis. The average time to recall is 30.46 months. The other demographic characteristics of the patient pool are summarized in Table 1.

**Table 1:** Population demographics

Demographic	Sealer extrusion	
	Absent, n(%)	Present, n(%)
**Total**	113 (61.1)	72 (38.9)
**Age (yrs)**		
<40	59 (31.9)	51 (27.6)
40–70	54 (29.2)	21 (11.3)
**Sex**		
Male	22 (11.9)	14 (7.6)
Female	91 (49.2)	58 (31.3)
**Tooth type**		
Anterior	34 (18.4)	27 (14.6)
posterior	79 (42.7)	45 (24.3)
**Tooth location**		
Maxillary	74 (40.0)	42 (22.7)
Mandibular	39 (21.1)	30 (16.2)
**Time to recall (y)**		
1–2	40 (21.6)	29 (15.7)
2–4	54 (29.2)	31 (16.7)
> 4	19 (10.3)	12 (6.5)
**Average time** **to recall**	30.46	
**Treatment type**		
Initial RCT	86 (46.5)	55 (29.7)
ReTx	27 (14.6)	17 (9.2)

*RCT* Root canal treatment, *ReTx* Retreatment

The overall success rate of all teeth was 96.8%. The success rate of teeth without sealer extrusion was 97.3%, with 88.5% healed, 8.8% healing, and 2.7% not healed. The overall success rate for teeth with sealer extrusion was 95.8%, with 69.4% healed, 26.4% healing, and 4.2% unhealed. No significant difference was found between the two types. Table 2 shows the outcomes of the comparison of teeth with and without sealer extrusion according to the prognostic factors. None of the most common factors showed a significant influence on the outcome of the two types of treated teeth. Because no failure was found among the cases with or without sealer extrusion according to the tooth type (anterior), the success rates in both groups were 100% and could not be compared. The exact numbers can be found in Table 2.

**Table 2:** Relation of prognostic factors to treatment results of teeth with or without sealer extrusion in root canals filled with gutta-percha and iRoot SP

Factor	Without sealer extrusion				With sealer extrusion				***P*** value
	Healed, n(%)	Healing, n(%)	Not healed, n(%)	Success, n(%)	Healed, n(%)	Healing, n(%)	Not healed, n(%)	Success, n(%)	
**Total**	100 (88.5)	10 (8.8)	3 (2.7)	110 (97.3)	50 (69.4)	19 (26.4)	3 (4.2)	69 (95.8)	0.679
**Age**									
<40	49 (83.0)	8 (13.6)	2 (3.4)	57 (96.6)	34 (66.7)	14 (27.4)	3 (5.9)	48 (94.1)	0.661
40–70	51 (94.4)	2 (3.7)	1 (1.9)	53 (98.1)	16 (76.2)	5 (23.8)	0 (0)	21 (100.0)	1.000
**Sex**									
Male	17 (77.3)	4 (18.2)	1 (4.5)	21 (95.5)	10 (71.4)	4 (28.6)	0 (0)	14 (100.0)	1.000
Female	83 (91.2)	6 (6.6)	2 (2.2)	89 (97.8)	40 (69.0)	15 (25.9)	3 (5.1)	55 (94.9)	0.378
**Tooth type**									
Anterior	29 (85.3)	5 (14.7)	0 (0)	34 (100.0)	19 (70.4)	8 (29.6)	0 (0)	27 (100.0)	**–**
posterior	71 (89.9)	5 (6.3)	3 (3.8)	76 (96.2)	31 (68.9)	11 (24.4)	3 (6.7)	42 (93.3)	0.667
**Tooth Location**									
Maxillary	66 (89.2)	7 (9.5)	1 (1.3)	73 (98.7)	32 (76.2)	10 (23.8)	0 (0)	42 (100.0)	1.000
Mandibular	34 (87.2)	3 (7.7)	2 (5.1)	37 (94.9)	18 (60.0)	9 (30.0)	3 (10.0)	27 (100.0)	0.646
**Time to recall**									
1–2	32 (80.0)	7 (17.5)	1 (2.5)	39 (97.5)	12 (41.4)	16 (55.2)	1 (3.4)	28 (96.6)	1.000
2–4	52 (96.2)	1 (1.9)	1 (1.9)	53 (98.1)	29 (93.6)	1 (3.2)	1 (3.2)	30 (96.8)	1.000
> 4	16 (84.2)	2 (10.5)	1 (5.3)	18 (94.7)	9 (75.0)	2 (16.7)	1 (8.3)	11 (91.3)	1.000
**Treatment type**									
Initial RCT	77 (89.5)	8 (9.3)	1 (1.2)	85 (98.8)	39 (70.9)	13 (23.6)	3 (5.5)	52 (94.5)	0.299
ReTx	23 (85.2)	2 (7.4)	2 (7.4)	25 (92.6)	11 (64.7)	6 (35.3)	0 (0)	17 (100.0)	0.515
**Lesion**									
Absent	57 (96.6)	0 (0)	2 (3.4)	57 (96.6)	27 (100.0)	0 (0)	0 (0)	27 (100.0)	1.000
Present	43 (79.6)	10 (18.5)	1 (1.9)	53 (98.1)	23 (51.1)	19 (42.2)	3 (6.7)	42 (93.3)	0.327
**Lesion size**									
≤ 5 mm	33 (82.5)	6 (15.0)	1 (2.5)	39 (97.5)	16 (72.8)	5 (22.7)	1 (4.5)	21 (95.5)	1.000
> 5 mm	10 (71.4)	4 (28.6)	0 (0)	14 (100.0)	7 (30.4)	14 (60.9)	2 (8.7)	21 (91.3)	0.517

We also analyzed whether the potential factors affected the success rate of the teeth with sealer extrusion, and the outcomes are shown in Table 3. All factors show no significant effect on the success rate of teeth with sealer extrusion. Among 72 extruding cases, none of the overfilling iRoot SP was completely absorbed during the recall time. 40.3% of them were partially absorbed and 59.7% appealed no absorption, showed in Table 4.

**Table 3:** Relation of prognostic factors to treatment results of teeth with sealer extrusion in root canals filled with gutta-percha and iRoot SP

Factor	Healed, n(%)	Healing, n(%)	Not healed, n(%)	Success, n(%)	***P*** value
**Age (yrs)**					0.551
<40	34 (66.7)	14 (27.4)	3 (5.9)	48 (94.1)	
40–70	16 (76.2)	5 (23.8)	0 (0)	21 (100.0)	
**Sex**					1.000
Male	10 (71.4)	4 (28.6)	0 (0)	14 (100.0)	
Female	40 (69.0)	15 (25.9)	3 (5.1)	55 (94.9)	
**Tooth type**					0.287
Anterior	19 (70.4)	8 (29.6)	0 (0)	27 (100.0)	
Posterior	31 (68.9)	11 (24.4)	3 (6.7)	42 (93.3)	
**Tooth Location**					0.068
Maxillary	32 (76.2)	10 (23.8)	0 (0)	42 (100.0)	
Mandibular	18 (60.0)	9 (30.0)	3 (10.0)	27 (100.0)	
**Time to recall (y)**					0.563
1–2	12 (41.4)	16 (55.2)	1 (3.4)	28 (96.6)	
2–4	29 (93.6)	1 (3.2)	1 (3.2)	30 (96.8)	
> 4	9 (75.0)	2 (16.7)	1 (8.3)	11 (91.3)	
**Treatment type**					1.000
Initial RCT	39 (70.9)	13 (23.6)	3 (5.5)	52 (94.5)	
ReTx	11 (64.7)	6 (35.3)	0 (0)	17 (100.0)	
**Lesion**					0.287
Absent	27 (100.0)	0 (0)	0 (0)	27 (100.0)	
Present	23 (51.1)	19 (42.2)	3 (6.7)	42 (93.3)	
**Lesion size**					1.000
≤ 5 mm	16 (72.8)	5 (22.7)	1 (4.5)	21 (95.5)	
> 5 mm	7 (30.4)	14 (60.9)	2 (8.7)	21 (91.3)	
**Extruded sealer size**					0.239
≤ 1 mm	29 (80.6)	7 (19.4)	0 (0)	36 (100.0)	
> 1 mm	21 (58.4)	12 (33.3)	3 (8.3)	33 (91.7)

**Table 4:** The absorption extent of iRoot SP in the teeth with sealer extrusion

Extent	Time to Recall (y)		
	1–2, n%	2–4, n%	> 4, n%
**Partial Absorption**	7 (9.7)	18 (25.0)	4 (5.6)
**No Absorption**	22 (30.5)	13 (18.1)	8 (11.1)

### Typical case

**Fig. 4:**
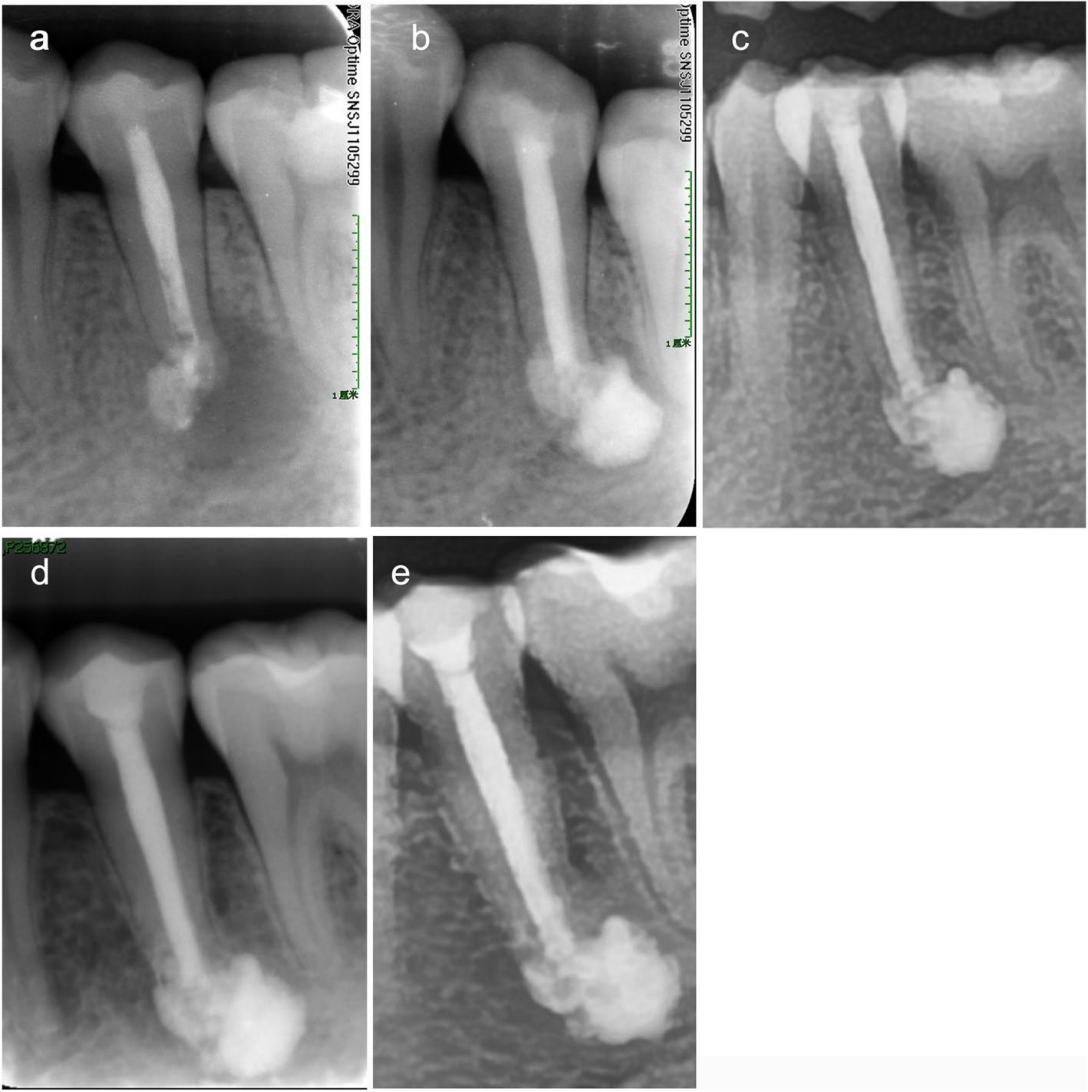
The radiographs of tooth #35. **a** The preoperative radiograph, showed that tooth #35 had large periapical lesion and overfilling intracanal medication. **b** The postoperative radiograph, showed that a larger amount of iRoot SP extruded. **c** The 12-month observation showed that periapical lesions were smaller according to the X-ray results. **d** The 36-month recall observation showed that periapical lesions disappeared radiographically. **e** Radiography results of 60-month following-up showed periapical lesions were invisible on tooth #35 and functioned without any symptoms. The over-filling iRoot SP appealed no absorption radiographically

A 31-year-old female patient complained that intermittent pain occurred in relation to the lower left posterior teeth over a month after treated in a dental clinic (Fig. 4). Clinical examination found pulp access cavity on tooth #35, with slight knocking pain. Diagnostic X-ray showed that tooth #35 had overfilling intracanal medication and large periapical lesion (Fig. 4a). According to the above results, we diagnosed the patient with chronic apical periodontitis. Endodontic retreatment was scheduled. After completed the root canal therapy of tooth #35, a larger amount of iRoot SP extruded due to the severe bone loss of periapical tissues (Fig. 4b). Subsequently, the tooth was reconstructed with direct composite resin. The 12-month recall observation showed that periapical lesions were smaller according to the X-ray results, without any symptoms (Fig. 4c). The radiographic follow-ups at 36 and 60 months showed periapical lesions disappeared and the over-filling iRoot SP appealed no obvious absorption radiographically (Fig. 4d-e).

## Discussion

In this study, a single-cone obturation technique with iRoot SP as the sealer was used for all of the cases, and the overall success rate of all teeth was 96.8%. This result coincided with the report of Chybowski et al. [33] that they followed 307 teeth with a single-cone technique for at least 1 year and found that the overall success rate was up to 90.9% compared to the cumulative success rate of 86% in the initial treatment with the vertically compacted warm gutta-percha technique [34]. This result demonstrates that the single-cone obturation technique is a viable option while sealers with superior properties remain in demand [33]. iRoot SP is a kind of bioceramic sealer with good biocompatibility, hydrophilicity and slight setting expansion, allowing it to be used in a single-cone technique. This technique enhances the clinical operation efficiency of root canal obturation and might even improve the success rate of endodontic therapy.

Within the limitations of this study, the overall success rate of adequately filled teeth was 97.3%, while that of iRoot SP extrusion was 95.8%; the difference was not statistically significant. More specifically, factors such as gender, age, tooth position, follow-up visit period, size of periapical lesions, and treatment type had no influence on the healing of the periapical tissues between the iRoot SP adequately filled group and the extrusion group. Further results showed that in the iRoot SP extrusion group, the factors mentioned before, including the amount of added extruding sealer, had little impact on the success rate of root canal treatment. These results were in contradiction with previous reports stating that extrusion of root filling material might interfere with the repair process [35–37]. However, Sari et al. [38] tracked 87 root canals for a 4-year follow-up period, demonstrating that extruded AH Plus does not prevent periapical healing but can be a delaying factor for healing in children. Zemener et al. [39] observed 10 cases that were overfilled with the methacrylate resin-based sealer EndoREZ and no adverse effect on the outcome. Ricucci et al. [40] found that all of the overextension teeth without periapical lesions healed within a 4-year follow-up period, while 79% of the overextension teeth with periapical lesions healed. These contradictions might be attributed to differences in tissue compatibility among the sealers. With the evolution of dental instruments and materials, bioceramic-based sealers have become widely used. Both Chang et al. [6] and Nagmode et al. [7] reported that the MTA extrusions in apexification did not affect the healing of periapical tissues and that the patients did not suffer. Asgary and Ehsani [8] reported a case in which extruding MTA was completely absorbed 7 years posttreatment and periapical tissues healed well. Nosrat et al. [41] reported 3 cases of MTA extrusions: MTA was absorbed in one case, and periapical healing was favorable after 4 years; in the other two cases, the teeth were persistently swelling and were sensitive to percussion. Thus, the outcome is unpredictable in the case of MTA overextension. Chybowski et al. [33] followed 307 teeth with a single-cone technique, and 47.4% of those teeth were iRoot SP overextended. After an average follow-up visit of 30.1 months, the results showed that iRoot SP extrusion had no significant influence on periapical tissue healing.

Our results, as well as those in a previous study [33], showed that iRoot SP extrusion has no adverse effect on the healing of periapical tissues. Its favorable properties might be one of the critical reasons. iRoot SP has been reported to be less toxic [10–17] than AH Plus and MTA and to induce a relatively milder inflammatory response [18]. Furthermore, the excellent apical sealing ability of iRoot SP also accounts for the favorable outcome, which results in less apical microleakage of bacteria [19–25]. Additionally, this ability plays a role in continuously inhibiting bacteria [28–32], as well as promoting osteogenesis after root canal obturation [13, 16, 18, 26, 27].

The overfilling sealers might dissolve in periapical tissue liquids and then be phagocytosed or wrapped by fibrous tissues. The outcome depends on the sealer’s composition and extrusion amount. Histological examination revealed that an obvious inflammatory response might occur in periapical tissues shortly after sealer extrusion [4]. However, except for several remarkably toxic sealers, once the sealers set, the toxicity vanishes. Consequently, there is no significant infection around the extruding sealer, but there is proliferation of fibrous connective tissue and sporadic infiltration of inflammatory cells [4].

In this study, among the iRoot SP extruding teeth, none presented complete absorption of extruding iRoot SP, whereas the periapical tissues healed or were healing. It is suggested that iRoot SP overextension is not the critical factor in periapical healing, which is in accord with the study of Lin et al. [42], who stated that extrusion of the sealer cannot be a factor in the failure of endodontic treatment and that the canal filler is likely less than microbial factors to cause irritation to periradicular tissue. In addition, sealer extrusion does not always lead to clinical symptoms nor is it the direct cause of postoperative pain, which might be associated with infection in root canals and periapical tissues. Moreover, sealer extrusion is likely to occur on those teeth with root apex absorption or canal over-preparation, which results in compromised sealing and microleakages at the apical foramen [43]. When the root canals are over-prepared, dental debris and necrotic pulp tissues containing bacteria might be pushed out into the periapical tissues. Under the above situations, bacteria transferred into the periapical tissues might multiply again and cause clinical symptoms. In addition, we don’t support the iRoot SP sealer extrusion because it may lead to facial paresthesia or maxillary sinusitis after the filling materials extrusion into the maxillary sinus or inferior alveolar canal [44, 45]. This article aims to further provide clinical data support to the operators, by reporting specifically on the clinical success of iRoot SP extrusion in root canal therapy and the potential factors affecting the outcomes. As a retrospective study, it is limited by bias. The inherent selection bias of retrospective studies might alter the outcome [46]. In this study, patients were regularly invited to recall visits, but many refused due to inconvenience. Thus, patients with symptoms were more willing to participate in subsequent visits, which decreased the success rate. In addition, our sample size was greatly reduced. Specifically, among the 99 patients, 78.8% were women. Women might pay more attention to their personal health than men do.

## Conclusion

Within the limitations of this study, the overall success rate of the iRoot SP extrusion group was 95.8% and was not statistically significant when compared to that of the iRoot SP adequately filled group. Factors such as gender, age, tooth position, follow-up visit period, size of periapical lesions, and treatment type had no influence on the healing of the periapical tissues between the iRoot SP adequately filled group and the extrusion group. In the iRoot SP extrusion group, the factors mentioned before with the addition of the extruding sealer amount had little impact on the success rate. To make our study more convincing, more follow-up recall cases and longer recall periods are needed in the future. Last, but not least, this study does not aim to advocate for sealer extrusion but suggests that operators could be optimistic when applying iRoot SP extrusion, except in special situations such as nerve injuries. The operators should pay more attention to root canal preparation and infection control.

## Data Availability

The datasets used and/or analyzed during the current study are available from the corresponding author on reasonable request.
